# Role of 5-aminolevulinic acid in the salinity stress response of the seeds and seedlings of the medicinal plant *Cassia obtusifolia* L.

**DOI:** 10.1186/1999-3110-54-18

**Published:** 2013-08-23

**Authors:** Chun-Ping Zhang, Yi-Cun Li, Feng-Gang Yuan, Shi-Jun Hu, Hai-Ying Liu, Ping He

**Affiliations:** 1 School of Life Sciences, Southwest University, Key Laboratory (Ministry of Education) of Eco-environments of Three Gorges Reservoir Region, Chongqing Key Laboratory of Plant Ecology and Resources Research for Three Gorges Reservoir Region, Chongqing, 400715 PR China; 2 Department of Biology and Center for Chinese Medicine Research, Hong Kong University of Science and Technology, Hong Kong SAR, 999077 PR China; 3 Laboratory Center, Xuzhou Medical College, Xuzhou, 221002 PR China; 4 School of Forestry, Southwest Forestry University, Kunming, 650224 PR China

**Keywords:** 5-Aminolevulinic acid, Antioxidant enzymes, *Cassia obtusifolia* L., Chlorophyll, Chlorophyll fluorescence, Salinity stress, Seed germination

## Abstract

**Background:**

Soil salinity, one of the major abiotic stresses affecting germination, crop growth, and productivity, is a common adverse environmental factor. The possibility of enhancing the salinity stress tolerance of *Cassia obtusifolia* L. seeds and seedlings by the exogenous application of 5-aminolevulinic acid (ALA) was investigated.

**Result:**

To improve the salinity tolerance of seeds, ALA was applied in various concentrations (5, 10, 15, and 20 mg/L). To improve the salinity tolerance of seedlings, ALA was applied in various concentrations (10, 25, 50, and 100 mg/L). After 10 mg/L ALA treatment, physiological indices of seed germination (i.e., germination vigor, germination rate, germination index, and vigor index) significantly improved. At 25 mg/L ALA, there was a significant protection against salinity stress compared with non-ALA-treated seedlings. Chlorophyll content, total soluble sugars, free proline, and soluble protein contents were significantly enhanced. Increased thiobarbituric acid reactive species and membrane permeability levels were also inhibited with the ALA treatment. With the treatments of ALA, the levels of chlorophyll fluorescence parameters, i.e., the photochemical efficiency of photosystem II (*F*_v_*/F*_m_), photochemical efficiency (*F*_v_'*/F*_m_'), PSII actual photochemical efficiency (ΦPSII), and photochemical quench coefficient (*qP*), all significantly increased. In contrast, the non-photochemical quenching coefficient (NPQ) decreased. ALA treatment also enhanced the activities of superoxide dismutase, peroxidase, and catalase in seedling leaves. The highest salinity tolerance was obtained at 25 mg/L ALA treatment.

**Conclusion:**

The plant growth regulator ALA could be effectively used to protect *C. obtusifolia* seeds and seedlings from the damaging effects of salinity stress without adversely affecting plant growth.

**Electronic supplementary material:**

The online version of this article (doi:10.1186/1999-3110-54-18) contains supplementary material, which is available to authorized users.

## Background

Soil salinity has become a global problem. A large number of lands are being eroded by salt, and numerous plants are being subjected to increasing salinity stress. Soil salinity, one of the major abiotic stresses affecting germination, crop growth, and productivity, is a common adverse environmental factor. Soil salinity affects plant growth, the global geographic distribution of vegetation, and the restriction of medicinal plant yields (Zhang et al. 2011). Under salinity stress, plants are very adversely affected by the generation of harmful oxygen species, leading to oxidative stress (Ahmad et al. 2005; Wahid et al. 2007).

Several protective mechanisms change to different extents with increased amounts of oxygen free radicals. Such mechanisms include those involving free radical and peroxide scavenging enzymes, e.g., superoxide dismutase (SOD), peroxidase (POD), and catalase (CAT) (McDonald 1999; Li et al. 2008). SOD is key to the regulation of the amounts of superoxide radicals and peroxides. Hydrogen peroxide (H_2_O_2_) can form hydroxyl radicals via the Haber-Weiss reaction, subsequently causing lipid peroxidation. CAT and POD are implicated in the removal of H_2_O_2_ (Zhang et al. 2010). H_2_O_2_ removal via a series of reactions is known as the ascorbate glutathione cycle. In this cycle, ascorbate and glutathione participate in a cyclic transfer of reducing equivalents resulting in the reduction of H_2_O_2_ to H_2_O using electrons derived form nicotinamide adenine dinucleotide phosphate (Goel and Sheoran 2003). The germination vigor and rate of seed are also reduced under salt stress. Some other symptoms of salinity stress include malondialdehyde increase, protein degradation.

Chl fluorescence is widely used in analyzing photosynthetic apparatuses. Chl fluorescence is also employed to understand the mechanism of photosynthesis and the mechanism by which a range of environmental factors alter photosynthetic activity under both biotic and abiotic stresses (Sayed 2003). Fluorescence parameters have also been applied in the rapid identification of injury to leaves in the absence of visible symptoms, and in the detailed analysis of change in photosynthetic capacity (Maxwell and Johnson 2000). Therefore, Chl fluorescence may be used as a potential indicator of environmental stress and a screening method of stress-resistant plants.

5-Aminolevulinic acid (ALA) is a key precursor in the biosynthesis of all porphyrins compounds, such as Chl, heme, and phytochrome (Eiji et al. 2003). The exogenous applications of ALA regulate plant growth and development, as well as enhance Chl biosynthesis and photosynthesis, resulting in increased crop yield (Hotta et al. 1997a). In plants, ALA concentration is strictly controlled to less than 50 nmol·g^-1^ FW (Stobart and Ameen-Bukhari 1984). ALA undergoes enolization and further metal-catalyzed aerobic oxidation at physiological pH to yield superoxide radical (O_2_^-^·), hydrogen peroxide (H_2_O_2_), and hydroxyl radical (HO·). Accumulated Chl intermediates are assumed to act as photosensitizers for the formation of singlet oxygen (^1^O_2_), triggering photodynamic damage in ALA-treated plants (Chakrabory and Tripathy 1992). Therefore, ALA accumulation enhances the levels of reactive oxygen species (ROS), leading to oxidative stress and herbicidal activity. Herbicidal activity has been reported to increase the accumulation of several Chl intermediates, such as protochlorophyllide, protoporphyrin IX, and Mg-protoporphyrin IX, when plants are treated with exogenous ALA at relatively high concentrations (5 mmol·L^-1^ to 40 mmol·L^-1^). However, low ALA concentrations (0.06 mmol·L^-1^ to 0.60 mmol·L^-1^) appear to promote rather than damage plant growth by increasing nitrate reductase activity, increasing fixation of CO_2_ in light, and suppressing the release of CO_2_ in darkness (Hotta et al. 1997b). ALA treatments of rice, barley, potato, and garlic plants at their early growth stages promote plant growth and photosynthetic rates, resulting in significant yield enhancements. Low-concentration ALA applications are also known to enhance plant tolerance to cold (Wang et al. 2003) and salinity stresses (Nishihara et al. 2003; Zhang et al. 2006). At concentrations over 5 mmol·L^-1^, herbicidal effects are exhibited (Kumar et al. 1999), suggesting the great potential of ALA as a new non-toxic endogenous substance for agricultural applications (Wang et al. 2003).

*Cassia obtusifolia* L. is a well known traditional Chinese medicinal plant belonging to the medically and economically important family Leguminosae (Syn. Fabaceae), subfamily Caesalpinioideae (Joshi and Kapoor 2003). The seed of the plant, called Juemingzi in China, is widely used for treating headache, dizziness, as well as red and teared eyes. In previous investigations of this plant, a number of constituents have been isolated, including anthraquinones, anthrones, flavonoids, triterpenoids, and so on. The anthraquinone derivatives, anthronic, dianthronic, and anthraquinone glycosides of *Cassia* are responsible for its purgative action (Anu and Rao 2001 ).

The mechanisms of ALA in promoting stress tolerance in plants need to be elucidated. The present paper provides the first evidence of the capability of ALA to protect *C. obtusifolia* seeds and seedlings against salinity stress, significantly contributing to the understanding of the ALA role in promoting salinity stress tolerance. The specific objective is to determine the optimum ALA concentration that provides the best protection against this stress.

## Methods

### Chemicals

ALA was obtained from the Korea Advanced Institute of Science and Technology (KAIST, Korea). All other chemicals were of analytical grade and obtained from Sigma Chemical (St. Louis, Missouri, U.S.A).

### Plant material

*C. obtusifolia* seeds were obtained from Institute of Medicinal Plant Development, Chinese Academy of Medical Sciences (Beijing, P.R. China). The seeds were surface sterilized with 2% (v/v) sodium hypochlorite solution for 10 min, and thoroughly washed with distilled water (Korkmaz 2005). The seeds were then germinated in covered 9 cm Petri dishes. Based on a preliminary experiment, 100 mmol·L^-1^ NaCl was used in the salinity stress experiment.

### Seed germination treatments

The seed germination experiment included seven treatments: (1) distilled deionized water (CK1), (2) 100 mmol·L^-1^ NaCl (CK2), (3) 10 mg·L^-1^ ALA (CK3), (4) 100 mmol·L^-1^ NaCl +5 mg· L^-1^ ALA (T1), (5) 100 mmol·L^-1^ NaCl +10 mg·L^-1^ ALA (T2), (6) 100 mmol·L^-1^ NaCl +15 mg·L^-1^ ALA (T3), and (7) 100 mmol·L^-1^ NaCl +20 mg·L^-1^ ALA (T4). Water was supplemented to the dishes every day and three replicates with fifty seeds per dish were used for each treatment in a light incubator under a 12/12 h photoperiod (light/dark; 450 μmol·m^-2^·s^-1^; 25 ± 1°C). The experiment was repeated three times under the same conditions (n=9). Radicle emergence was the criterion used to assess germination, and was recorded daily for 6 d until the numbers stabilized. Germinated seeds were removed from the Petri dishes. The physiological indices of seed germination were determined as follow: germination vigor (GV) = *A*/*C*, germination rate (GR) = *B*/*C*, germination index (GI) = Σ(*Gt*/*Dt*), and vigor index (VI) = GI × *S*. *A* is the total number of seeds germinated in 4 d, *B* is the total number of seeds germinated in 6 d, and *C* is the total seeds in the experiment. *Gt* is the germination percentage after *t* days, and *Dt* is the days of germination. *S* is the radicle mean and length upon the termination of germination (6 d later). The fresh weight as well as length of radicle and plumule with different treatments were measured after the germination stopped.

### Seedlings treatments

Seeds without any treatment were sown in pots filled with growth medium consisting of 4:1 peat and perlite. The pots were watered and placed in a greenhouse under a 12/12 h photoperiod, 25/20°C (light/dark) temperature regime and 65% relative humidity. At the two-real-leaf stage, seedlings were irrigated with half-strength of Hoagland nutrient solution every day. After 25 d of pre-culture, the seedlings were at the stage of 4 to 5 real leaves and the treatment was started. The seedling experiment included seven treatments: (1) Hoagland nutrient solution (CK1), (2) Hoagland nutrient solution + 100 mmol·L^-1^ NaCl (CK2), (3) Hoagland nutrient solution + 25 mg·L^-1^ ALA (CK3), (4) Hoagland nutrient solution + 100 mmol·L^-1^ NaCl + 10 mg·L^-1^ ALA (T1), (5) Hoagland nutrient solution + 100 mmol·L^-1^ NaCl + 25 mg·L^-1^ ALA (T2), (6) Hoagland nutrient solution + 100 mmol·L^-1^ NaCl + 50 mg·L^-1^ ALA (T3), and (7) Hoagland nutrient solution + 100 mmol·L^-1^ NaCl + 100 mg·L^-1^ ALA (T4). The nutrient solution was supplemented to the pots every other day, whereas the ALA was supplemented every day. The experimental design was a randomized complete block with six treatments arranged in individual pots with nine plants per treatment and three replicates each. The experiment was repeated three times under the same conditions (n=9).The experiment with all treatment were executed at dusk because ALA easily decomposes in light. The seedling samples were collected for assays at the 4th, 8th, and 12th days, respectively.

### Chlorophyll content determination

The contents of total Chl, Chl *a* and Chl *b* were determined by collecting fresh leaf samples (0.5 g) from randomly selected 9 plants per replicate. The samples were homogenized with 5 ml of acetone (80%, v/v) using a mortar and pestle before being filtered through a Whatman No.2 filter paper. The absorbance was measured using a UV-visible spectrophotometer (UV-2550, Shimadzu, Japan) at 663 and 645nm (Lichtenthaler 1987).

### Chlorophyll fluorescence

The Chl fluorescence of leaves was measured at room temperature (25°C) using a pulse-modulated fluorometer (PAM-2500, Walz, Germany) after the leaves were dark adapted for 30 min. The detailed experimental protocol was as followed (Genty et al. 1989). The minimal fluorescence *(F*_o_) with all PSII reaction centers open was measured with modulated light that was sufficiently low (<0.05 μmol·m^-2^·s^-1^ as not to induce significant variable fluorescence. The maximal fluorescence (*F*_m_) with all PSII reaction centers closed was determined by a 0.8 s saturating pulse at 8000 μmol·m^-2^·s^-1^ in dark-adapted leaves. Then, leaves were continuously illuminated with 336 μmol·m^-2^·s^-1^ white actinic light. The steady-state value of fluorescence (*F*_s_) was thereafter recorded and a second saturating pulse at 8000 μmol·m^-2^·s^-1^ was imposed to determine the maximal fluorescence in the light-adapted state (*F*_m_'). *F*_o_' was the basal fluorescence after far-red illumination. The photosynthetic parameters were calculated using the PAMWIN Data Acquisition System (Walz, Germany) as follow: the photochemical efficiency of PSII (*F*_v_/*F*_m_) = (*F*_m_*−F*_o_)/*F*_m_, the excitation capture efficiency of open PSII reaction centers (*F*_v_'/*F*_m_') = (*F*_m_'*−F*_o_')/*F*_m_', and the coefficients of photochemical quenching (*qP*) = (*F*_m_'*−F*_s_)/(*F*_m_'*−F*_o_'), coefficient of non-photochemical quenching (NPQ) *=* (*F*_m_*−F*_m_')/*F*_m_', and actual photochemical efficiency of PSII (ΦPSII*) =* (*F*_m_'*−F*_s_)/*F*_m_' (Demmig-Adams et al. 1996). Fluorescence measurements were performed in four-flag-leaf stage per treatment combination and all measurements were made between 8:00 to 11:00 a.m. and replicated at least six times.

### Membrane permeability determination

The level of membrane permeability was represented by the relative conductivity. The electrical conductivity of leaf leachates in double distilled water was recorded at 40 and 100°C (Sairam 1994). Leaf samples (0.1 g) were cut into uniformly sized discs and placed in test tubes containing 10 mL of double distilled water in two sets. One set was kept at 40°C for 60 min, and the other was at 100°C in boiling water bath for 30 min. Their respective electric conductivities *C*_1_ and *C*_2_ were measured by a conductivity meter. The relative conductivity index was (*C*_1_/*C*_2_) × 100%.

### Determination of thiobarbituric acid (TBA)-reactive substances

The level of lipid peroxidation was measured in terms of the content of TBA-reactive substances (TBARS) (Heath and Packer 1968). Fresh leaf samples (0.5 g) were homogenized in 10 mL of 0.1% trichloro-acetic acid (TCA). The homogenate was centrifuged at 15 000×*g* for 5 min. About 2 mL of aliquot of the supernatant was mixed with 4 mL of 0.5% TBA in 20% TCA. The mixture was heated at 95°C for 30 min, and then quickly cooled in an ice bath. After centrifugation at 10 000×*g* for 10 min to remove suspended turbidity, the absorbance of the supernatant was recorded at 532 nm. The value for nonspecific absorption at 600 nm was subtracted. The TBARS content was calculated using its absorption coefficient of 155·mmol^-1^·cm^-1^.

### Osmotic substances

The osmotic substances measured in the experiment included total soluble sugars, free proline, and soluble protein. Total soluble sugars were estimated using anthrone reagent (Yemm and Willis 1954). Samples were extracted with 4 ml of 80% methanol at 80°C for 40 min and were then centrifuged at 2000×*g* for 15 min. The methanol supernatants of three successive centrifugations were used for the sugar analyses. About 4 mL of anthrone reagent was then added. The mixture was heated in a boiling water bath for 8 min, and then cooled. Optical density of green to dark green color was read at 630 nm.

Free proline accumulation is widely used as a parameter of salt stress tolerance (Storey and Wyn-Jones 1975). In the present study, free proline content in leaves was determined by the following method. Fresh leaf samples (0.5 g) were homogenized in 5 mL of sulfosalicylic acid (3%) using a mortar and pestle. About 2 mL of the extract was placed in a test tube. About 2 mL each of glacial acetic acid and ninhydrin were added. The reaction mixture was boiled in a water bath at 100°C for 30 min. The reaction mixture was cooled, mixed with 6 mL of toluene, and then transferred into a separating funnel. After thorough mixing, the chromophore containing toluene was separated, and absorbance was read at 520 nm against a toluene blank. The concentration of free proline was estimated by referring to a standard curve (Bates et al. 1973).

Soluble protein was extracted from 0.5 g of fresh leaf using 2 mL of 50 mmol·L^-1^ sodium phosphate buffer (pH 7.4). Soluble protein content was determined by the method of using bovine serum albumin (BSA) as a standard (Lowry et al. 1951).

### Enzyme extraction and enzymatic activity determination

Fresh leaf samples (1.0 g) were rapidly extracted in a pre-chilled mortar on an ice bath using 5 ml of ice-cold 100 mmol·L^-1^ phosphate buffer (pH 7.8) containing 1.0 mmol·L^-1^ ethylenediaminetetraacetic acid and 5% (w·v^-1^) polyvinylpyrrolidone. After centrifugation at 12 000×*g* for 30 min at 4°C, the supernatant was used for the determination of enzymatic activities.

SOD activity was determined by first mixing 0.1 mL of the enzyme extract with 2.465 mL of 100 mmol·L^-1^ phosphate buffer (pH 7.8), 75 μL of 55 mmol·L^-1^ methionine, 300 μL of 0.75 mmol·L^-1^ nitroblue tetrazolium (NBT), and 60 μL of 0.1 mmol·L^-1^ riboflavin in a test tube. The test tubes containing the reaction solution were irradiated under 2 fluorescent light tubes (40 μmol·m^-2^·s^-1^) for 10 min. The absorbance was measured at 560 nm. Blanks and controls were run in the same manner but without illumination and the enzyme extract, respectively. One unit of SOD activity was defined as the amount of enzyme that inhibits 50% of NBT photo reduction (Xu et al. 2008).

POD activity was determined as follow. The reaction mixture contained 0.1 mL of enzyme extract, 2 mL of 0.1 mol sodium acetate buffer (pH = 4.5), and 0.5 mL of *o*-dianisidine solution (0.2% in methanol, freshly prepared). The reaction was initiated with the addition of 0.1 mL of 0.2 mol H_2_O_2_. The change in absorbance was recorded at 470 nm at an interval of 15 s for 2 min. One unit of POD was defined as 0.1 ΔA_470_/min (Kalpana and Madhava Rao 1995).

CAT activity was estimated as follow. The reaction mixture contained 0.6 mL enzyme extract, 0.1 mL of 10 mmol H_2_O_2_, and 2 mL of 30 mmol phosphate buffer (pH = 7.0). The absorbance was recorded at 240 nm immediately after enzyme extract addition at an interval of 15 s for 2 min. The blank did not contain enzyme extract. One unit of CAT was defined as 0.1 Δ*A*_240_/min (Goel and Sheoran 2003).

### Statistics

Values were presented as the mean ± standard deviation of there replicates. Statistical analyses were carried out by ANOVA. Tukey's test was used to compare the means multiple treatments. To fit the normality, percentage values were arcsine transformed prior to statistical analysis. The significance level was set at *P* = 0.05.

## Results

### Seeds germination

ALA with different concentrations significantly affected the seed germination indices GV, GR, GI, and VI of *C. obtusifolia* seeds under salinity stress (Table 1). The indices of seeds treated with only NaCl (CK2) significantly differed from the control (CK1).The indices improved after the treatment of ALA (CK3, 10 mg·L^-1^)only. On the other hand, the indices varied with increased ALA concentrations. The indices improved with different ALA concentrations (T1-T4), and treatment T2 raised the indices to a level that did not differ significantly from the unstressed control (CK1). The optimum ALA concentration for alleviating *C. obtusifolia* seed damage was 10 mg·L^-1^.

**Table 1:** **Germination vigor (GV), germination rate (GR), germination index (GI), and vigor index (VI) of** ***C. obtusifolia*** **seeds treated with different ALA under salinity stress** ^**a**^

Treatment	GV	GR	GI	VI
CK1	76.31 ± 4.01a	98.24 ± 7.01a	89.94 ± 3.01a	11.37 ± 1.12a
CK2	52.78 ± 7.23c	71.29 ± 7.34d	48.66 ± 5.32d	6.38 ± 0.83d
CK3	79.96 ± 5.49 a	99.12 ± 6.69 a	90.15 ± 4.98 a	11.52 ± 0.98 a
T1	60.44 ± 6.13b	81.33 ± 6.23b	75.36 ± 4.13b	8.03 ± 1.24b
T2	78.28 ± 6.15a	98.16 ± 8.19a	89.07 ± 6.15a	10.86 ± 1.39a
T3	65.32 ± 6.29ab	83.27 ± 5.46ab	80.42 ± 4.08ab	8.82 ± 1.27ab
T4	59.68 ± 6.23b	78.31 ± 8.44c	69.89 ± 5.53c	8.02 ± 1.44c

^a^Data are the means of three independent replicates. Means ± standard errors (*n* = 9) within each column followed by different letters are significantly different at the *P =* 0.05 level.

### Seedlings growth

The radicle length, plumule length, radicle/plumule ratio, and fresh weight of *C. obtusifolia* seedlings under salinity stress treated with different ALA concentrations were measured after germination was stopped. The tendencies of the radicle length, plumule length, and fresh weight with different treatments were similar (Table 2). Treatment with only NaCl stress (CK2) resulted in a significant decrease compared with no NaCl stress treatment (CK1) and reached the minimum value. However, different ALA concentrations improved the growth indices. The radicle/plumule ratio increased with all ALL treatments and treatments T2-T4 restored the parameter to values that did not differ significantly from the unstressed CK1. Under salinity stress, plumule growth was inhibited to a greater extent than radicle growth. The radicle/plumule ratio increased, possibly indicating that the different vegetative organs of seedlings adjust this ratio to ensure maximum survival and seedling growth.

**Table 2:** **Radicle length, plumule length, radicle/plumule ratio, and fresh weight of** ***C. obtusifolia*** **seedlings treated with different ALA concentrations under salinity stress after germination is terminated** ^**a**^

Treatment	Radicle length (cm)	Plumule length (cm)	Radicle/plumule ratio	Fresh weight (mg)
CK1	2.79 ± 0.36a	4.12 ± 0.65a	0.67 ± 0.13c	162.7 ± 5.7a
CK2	2.33 ± 0.25c	2.51 ± 0.68d	0.93 ± 0.16a	113.6 ± 6.4d
CK3	2.81 ± 0.43a	4.14 ± 0.66a	0.68 ± 0.16c	165.58 ± 5.2a
T1	2.52 ± 0.34b	3.56 ± 0.75b	0.71 ± 0.09bc	140.3 ± 3.5b
T2	2.78 ± 0.31a	4.13 ± 0.49a	0.67 ± 0.21c	161.5 ± 4.8a
T3	2.65 ± 0.18ab	3.74 ± 0.66ab	0.71 ± 0..6bc	148.8 ± 3.1ab
T4	2.60 ± 0.76ab	3.27 ± 0.53c	0.79 ± 0.08b	130.6 ± 4.4c

^a^Data are the means of three independent replicates. Means ± standard errors (*n* = 9) within each column followed by different letters are significantly different at the *P =* 0.05 level.

### Chl content

**Figure 1:**
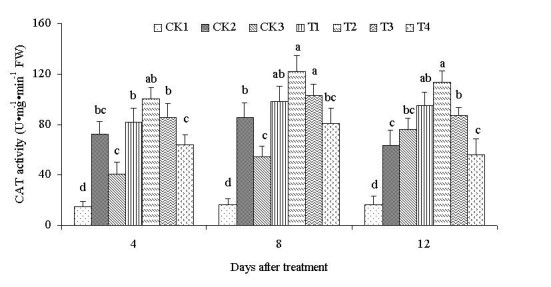
**The contents of chlorophyll** ***a*** **(A), chlorophyll** ***b*** **(B), and total chlorophyll (C) treated with different ALA concentrations under salinity stress.** Vertical bars indicate standard errors (*n* = 9). Mean values with different letters are significantly different by the Tukey test at the *P =* 0.05 level.

The seedling leaves of *C. obtusifolia* treated under NaCl stress (100 mmol/L) had lower Chl content than the control CK1 (Figure 1). Chl *a*, Chl *b*, and total Chl content (CK1, CK3) decreased with the treatment days compared with CK2. ALA applied in increasing concentrations to salinity stress expectedly enhanced the Chl content of the seedlings because ALA is a precursor in Chl biosynthesis. On the other hand, seedlings treated with increased ALA concentrations caused different enhancements in Chl content. ALA concentration increased with 25 mg·L^-1^ALA (T2) resulted in improved Chl content compared with the CK2. However, 10 mg/LALA has no significant difference compared with the CK2 at 4 and 8 days, further increased ALA concentration to 50 mg·L^-1^ (T3) caused a slight decrease of total Chl compared with 25 mg·L^-1^ (T2), and the minimum value was reached with 100 mg·L^-1^ ALA (T4).

### Chl fluorescence

**Figure 2:**
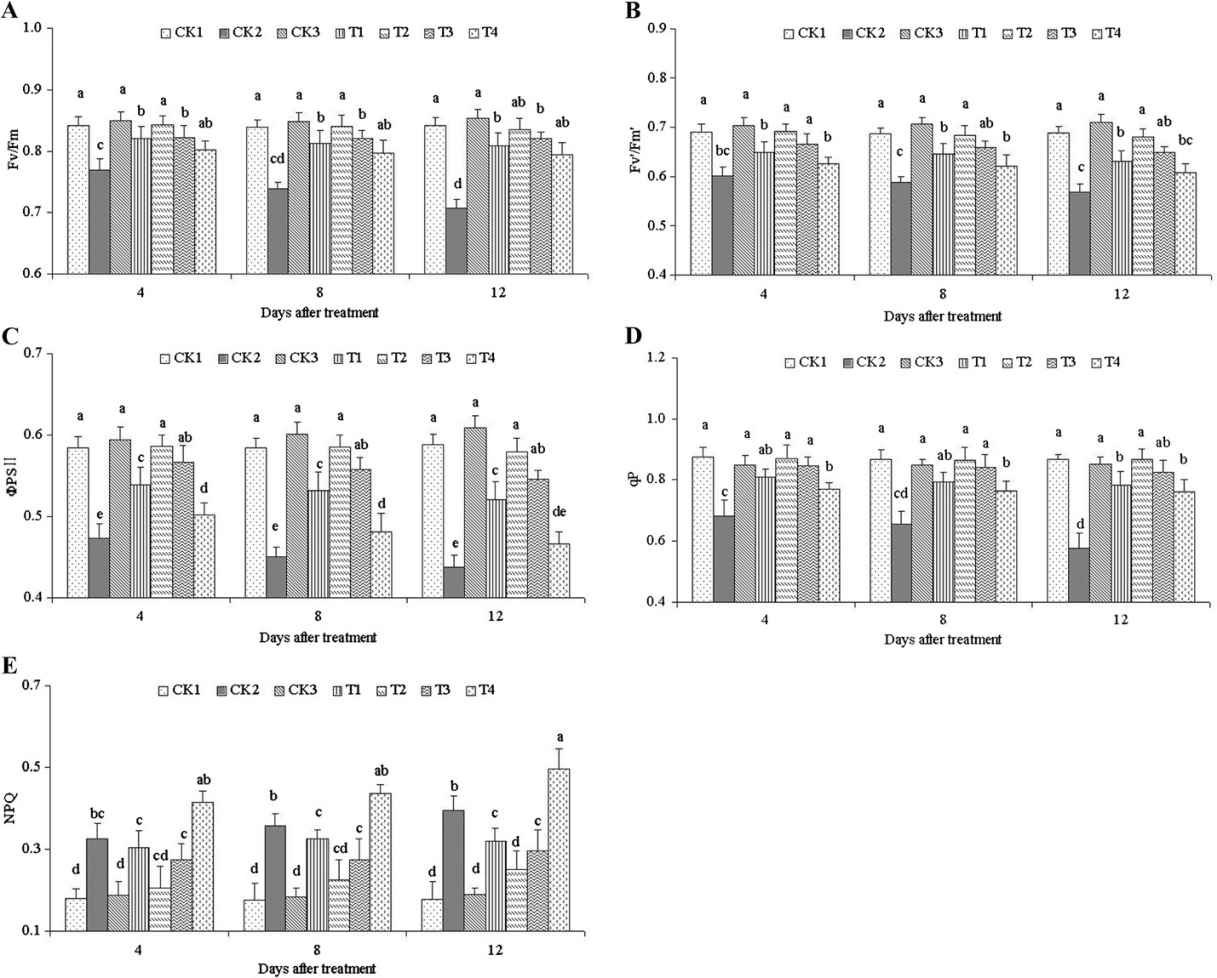
**Levels of** ***F***_**v**_**/** ***F***_**m**_**(A),** ***F***_**v**_**'/** ***F***_**m**_**' (B), ΦPSII (C),** ***qP*** **(D), and NPQ (E) treated with different ALA concentrations under salinity stress.** Vertical bars indicate standard errors (*n* = 9). Mean values with different letters are significantly different by the Tukey test at the *P =* 0.05 level.

The photochemical efficiency of PSII (*F*_v_*/F*_m_), the excitation capture efficiency of open PSII reaction centers (*F*_v_'/*F*_m_'), actual photochemical efficiency of PSII (ΦPSII), and coefficients of photochemical quenching (*qP*) under 100 mmol·L^-1^ NaCl stress (CK2) all significantly decreased compared with CK1 and CK3 (25 mg·L^-1^ ALA only), and the level increased with treatment days (Figure 2A–2D). However, after treatment with different ALA concentrations, these fluorescence parameters improved to various extents, and the differences were significant compared with CK2. The ALA concentration of 25 mg·L^-1^ at 4 d had the most significant effect and yielded the maximum values of 0.843 (*F*_v_*/F*_m_), 0.692 (*F*_v_'/*F*_m_'), 0.872 (*qP*) and 0.586 (ΦPSII). The change in the non-photochemical quenching (NPQ*)* was contrary; it increased with the 100 mmol·L^-1^ NaCl (CK2) treatment and decreased with different ALA concentrations (Figure 2E). The ALA concentration of 100 mg·L^-1^ had the opposite effect to the other parameters. Hence, the optimum ALA concentration was the relatively low 25 mg/L.

### Membrane permeability

**Figure 3:**
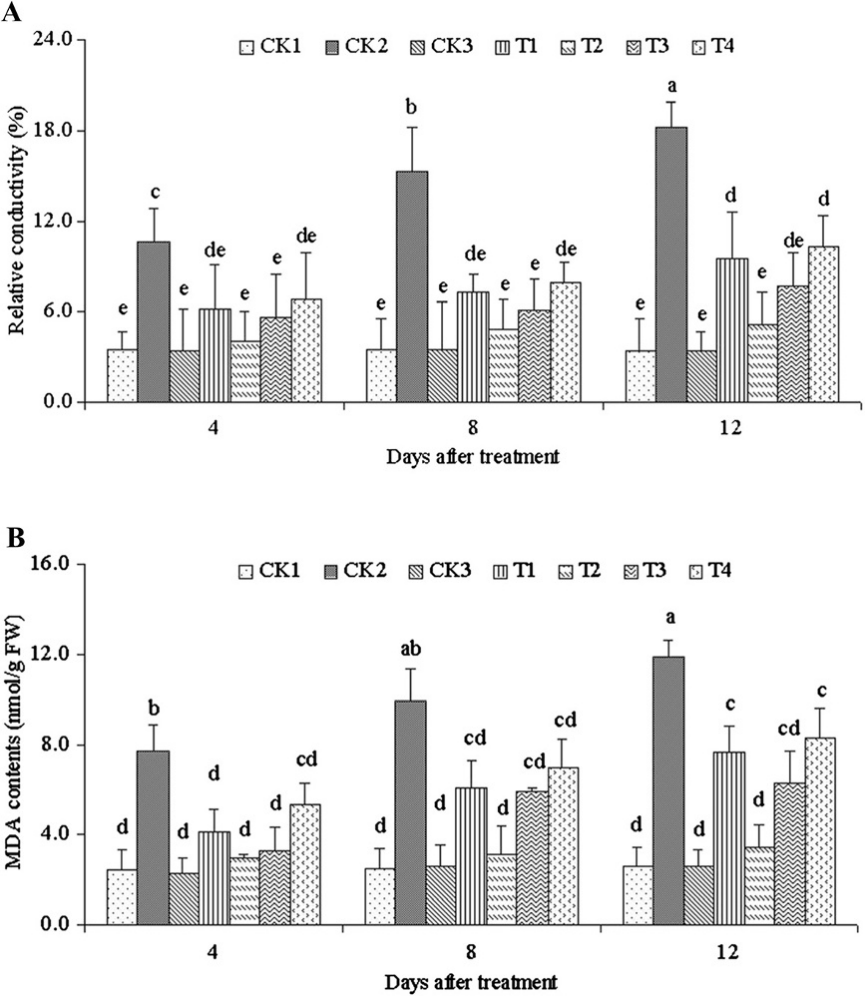
**Level of relative conductivity (A) and content of TBARS (B) treated with different ALA concentrations under salinity stress.** Vertical bars indicate standard errors (*n* = 9). Mean values with different letters are significantly different by the Tukey test at the *P =* 0.05 level.

The level of membrane permeability was represented by the relative conductivity. Figure 3A shows that the relative conductivity of the seedlings treated with only NaCl stress (CK2) significantly increased compared with that of the seedlings not treated with NaCl (CK1 and CK3). The maximum value of 18.17%, which was 5.31 times that of CK1 (3.42%), was reached 12 d later. However, the relative conductivity decreased with different ALA concentrations, and reached the minimum of 5.13% in the 25 mg·L^-1^ ALA treatment (T2) at 4 d. Treatment with ALA did not significantly differ from the control CK1. These results indicated that salinity stress resulted in the significantly increased membrane permeability of leaves. ALA treatment inhibited the increase in membrane permeability, and effectively decreased the salt stress on the cell plasma membrane damage.

### MDA

The results for lipid peroxidation, estimated as MDA content, are presented in Figure 3B. MDA content increased with treatment days under NaCl stress, and reached the maximum value on the 12th day with only 100 mmol·L^-1^ NaCl (CK2). The content significantly decreased with different ALA concentrations (T1–T4), and yielded the minimum value on the fourth day after treatment with 25 mg·L^-1^ ALA. All ALA treatments (T1–T4) had significant differences compared with the no ALA treatment (CK2).

### Total soluble sugars, free proline and soluble protein

**Figure 4:**
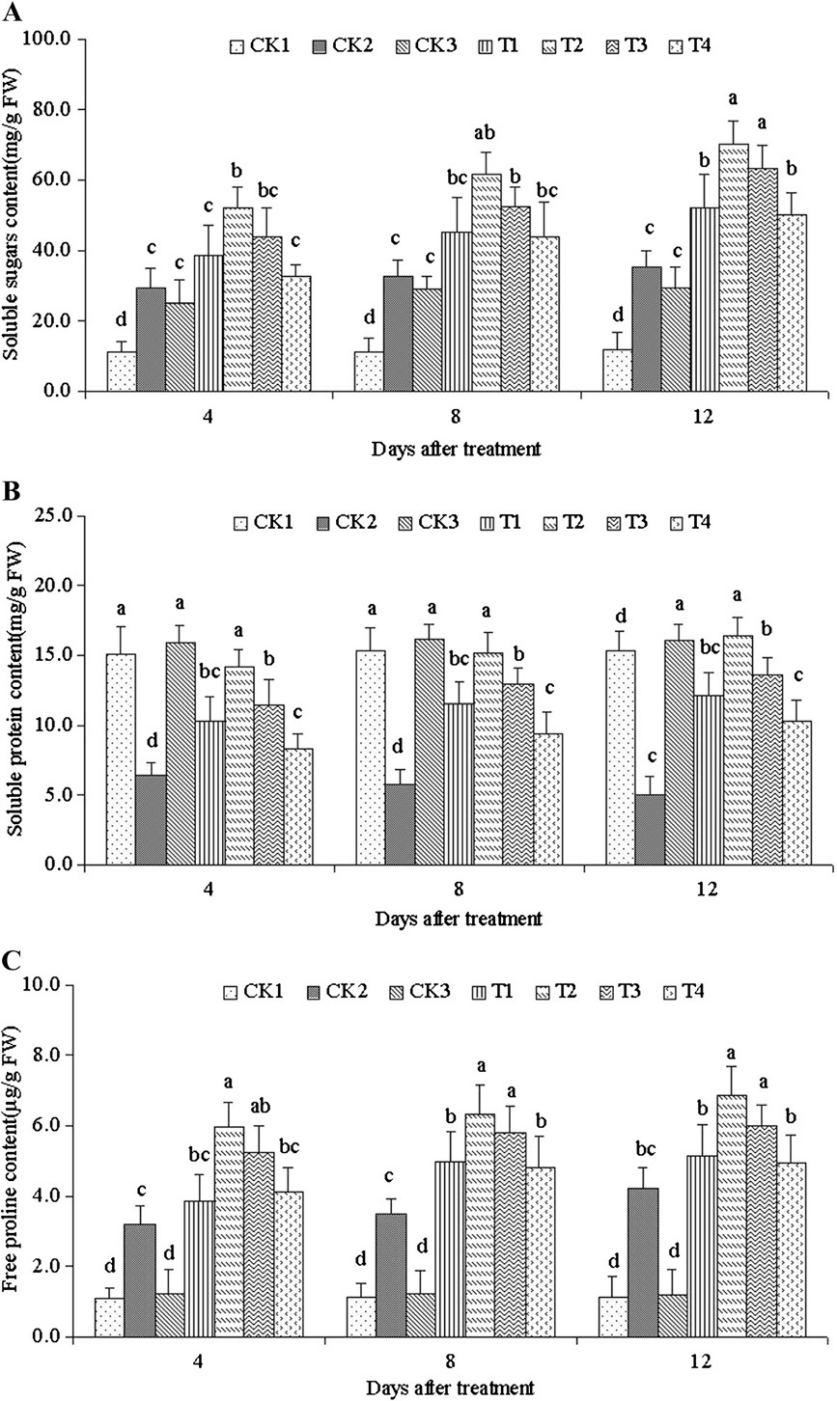
**Contents of soluble sugars (A), soluble protein (B), and free proline (C) treated with different ALA concentrations under salinity stress.** Vertical bars indicate standard errors (*n* = 9). Mean values with different letters are significantly different by the Tukey test at the *P =* 0.05 level.

The contents of total soluble sugars, soluble protein, and free proline under NaCl stress (CK2) all significantly increased compared with CK1 and CK3 (Figure 4). All contents increased in various degree after ALA treatment. All maximum values were reached at 12 d after 25 mg·L^-1^ ALA treatment. The soluble sugars and free proline content treated with different concentrations of ALA was similarly and the contents were all increased after the treatment (Figure 4A and 4B). The soluble protein content significantly decreased after the NaCl only treatment (CK2), and yielded the minimum value (Figure 4C). However, the soluble protein improved with different ALA concentrations, and reached the maximum value on the 12th day after treatment with 25 mg·L^-1^ ALA.

### Activities of three antioxidant enzymes

**Figure 5:**
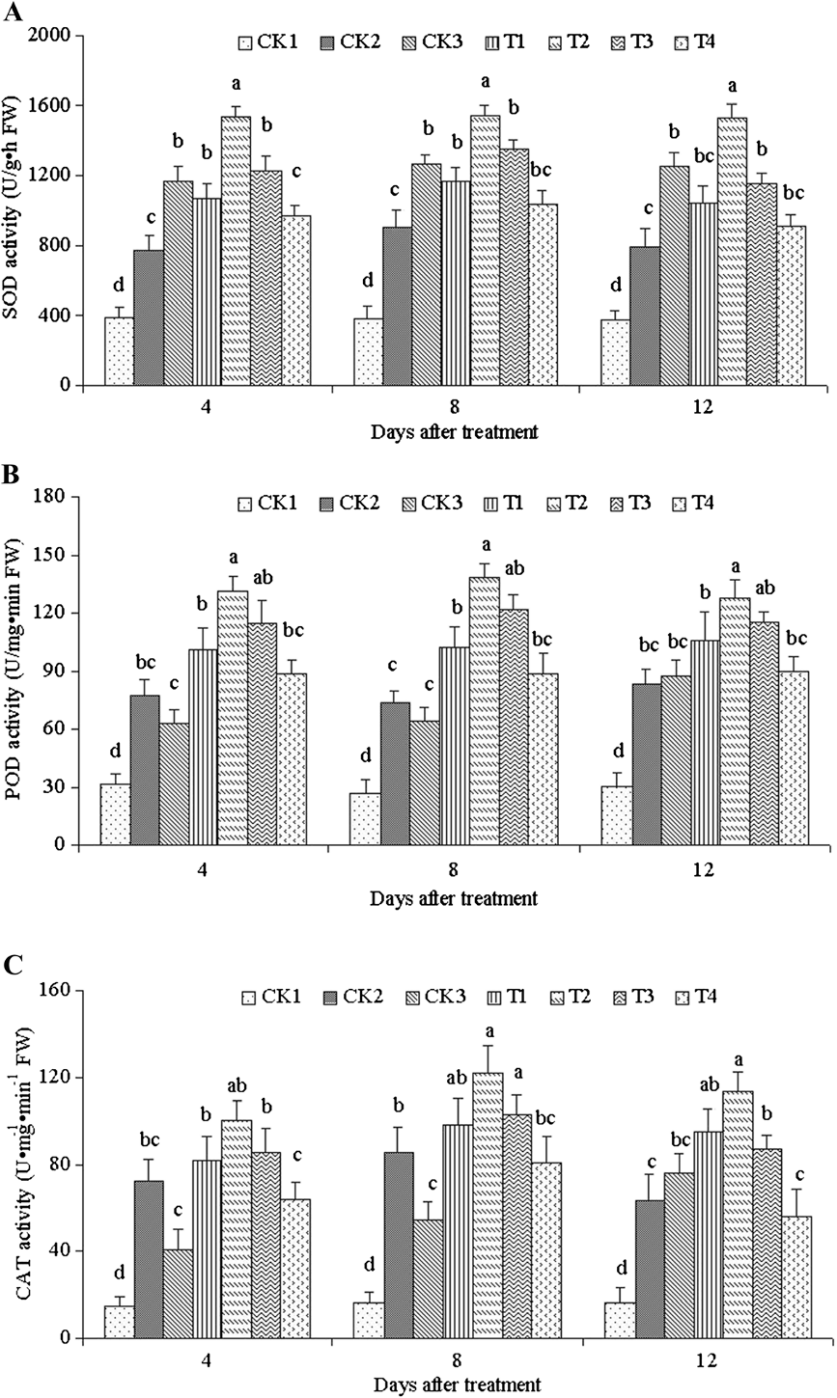
**Activities of SOD (A), POD (B), and CAT (C) treated with different ALA concentrations under salinity stress.** Vertical bars indicate standard errors (*n* = 9). Mean values with different letters are significantly different by the Tukey test at the *P =* 0.05 level.

To investigate further the action of ALA on salinity stress in *C. obtusifolia* plants, antioxidant enzyme activities were determined. The activities of SOD, POD, and CAT in response to ALA treatment under a 100 mmol·L^-1^ NaCl condition are shown in Figure 5. The activities of CAT, SOD, and POD increased after salinity stress (CK2, treated with only NaCl and no ALA) compared with the control CK1 (no treatment) and the control CK3 (25 mg·L^-1^ ALA and no NaCl). This result showed the obvious response of seedlings to stress. SOD activities treated with different ALA concentrations (T1–T4) significantly increased compared with CK1 and CK2 (Figure 5A). Over time, the activities of SOD initially increased, and then decreased. The maximum appeared 8 d after treatment. The activities under the treatments of 10, 25, 50, and 100 mg·L^-1^ ALA were strongly enhanced by 3.05-, 4.04-, 3.52-, and 2.70-fold respectively, compared with CK1 on the 8th day under a 100 mmol·L^-1^ NaCl condition, as well as by 1.29-, 1.71-, 1.49-, and 1.14-fold, respectively, compared with CK2. These results showed that different ALA concentrations improved the activity of SOD, and that the effect of 25 mg·L^-1^ ALA was the most significant. POD and CAT activities were similar with SOD and reached the maximum with 25 mg·L^-1^ ALA 8 d after treatment. The activities of POD and CAT were 138.11 and 122.04 U·mg^-1^·min^-1^, respectively. The various ALA treatments had significant differences from CK1 and CK2 (Figure 5B and 5C). However, the activities did not significantly differ at days 4, 8, and 12 after treatment. This finding indicated that the effect of ALA could be sustained for a long time under salinity stress.

## Discussion

Salinity is one of the most important abiotic stresses that affect crop productivity. Unlike drought, salinity stress is an intricate phenomenon that includes osmotic stress, specific ion effect, nutrient deficiency, etc. Consequently, salinity stress affects various physiological and biochemical mechanisms associated with plant growth and development. Plants have developed various combating mechanisms to cope with the deleterious effects of this stress.

Seed germination is a major stage in the life history of plant. It directly affects plant growth, development, and morphogenesis, as well as indirectly affects yield. Therefore, a seed that is able to germinate quickly is the foundation of high and stable yield. In the current study, the levels of GV, GR, GI, and VI of *C. obtusifolia* seeds were effectively improved by exogenous ALA application. The results showed that ALA improved the germination and emergence of *C. obtusifolia* seeds under salinity stress. ALA may have imbibed into the seeds during priming and imparted tolerance to salinity stress during seed germination and emergence. Hence, ALA treatment could increase the germination ability of *C. obtusifolia* seeds under NaCl stress.

Previous studies have shown that Chl can be bleached under oxidative stress (Noriega et al. 2004). These results can be explained in two ways. On one hand, ALA at low concentrations acts as a regulator of Chl and heme biosynthesis. On the other hand, oxidative stress may occur as a result of ROS generated by higher ALA concentrations. ALA, is the key precursor in the biosynthesis of all porphyrin compounds such as Chl and heme. ALA formation in plants is the rate-limiting step in tetrapyrrole biosynthesis (Von Wettstein et al. 1995). In the current paper, the Chl content of *C. obtusifolia* leaves greatly decreased after salinity stress, signifying that salinity stress injured the synthesis photosynthetic pigments. Treatment with low exogenous ALA concentrations (25 mg/L) enhanced Chl content compared with non-ALA-treated plants under salinity stress. Hence, the exogenous application of low-concentration ALA prior to salinity stress is a possible method for overcoming an inadequate biosynthesis problem.

Photosynthesis is an important metabolic process in plant and significantly affects plant growth, yield, as well as resistance to adverse environmental factors. Plant photosynthesis is obviously affected by salinity stress. The direct results are photosynthetic system damage, photophosphorylation, and photosynthetic electronic transfer. The indirect results are those involving enzymes in dark reactions. Consequently, photosynthesis can be used as a significant index for evaluating plant growth and tolerance. Chl fluorescence measurements have recently been used to estimate rapidly and non-invasively the operating quantum efficiency of electron transport via PSII in plant leaves (Baker and Rosenqvist 2004). The photochemical efficiency of PSII (*F*_v_/*F*_m_) is proportional to the potential maximal quantum yield of PSII (Hormann et al. 1994), *F*_v_/*F*_m_ is the efficiency of the primary conversion of the light energy of PSII, which indicates the ability of PSII light energy utilization and is closely related to the photoinhibition of the photosynthesis degree (Maxwell and Johnson 2000), *F*_v_/*F*_m_ is also called the optimal photochemical efficiency of PSII in the dark. *F*_v_'/*F*_m_' represents the light energy conversion efficiency of the open PSII center, which is called the effective photochemical efficiency or antenna pigment transformation efficiency of PSII in light (Zhang 1999). *F*_v_/*F*_m_ is one of the Chl fluorescence indices that are usually used in stress conditions. *F*_v_/*F*_m_ is decreased under stress, thereby indicating PSII damage (Xu and Zhang 1999). The actual photochemical efficiency of PSII (ΦPSII) reflects the actual original light energy-capturing efficiency of the PSII response center under some closed circumstances. In the current paper, Chl fluorescence kinetics indicated that *F*_v_/*F*_m_, *F*_v_'/*F*_m_', and ΦPSII significantly decreased under NaCl stress. This finding revealed that PSII suffered damage in various degrees. Nevertheless, the light energy capture efficiency significantly improved upon treatment with different ALA concentrations.

The coefficients of photochemical quenching (*qP*) could reflect the redox state of PSII original electronic receptor QAs and the number of PSII open centers. *qP* reflects the PSII centers of openness to some extent, and the non-photochemical quenching (NPQ) is the photosynthetic apparatus of self-protective mechanisms (Havaux et al. 1991). The NPQ process is an adaptive mechanism that prevents photoinhibition and membrane damage to plants by adjusting the dissipation of excessive energy. The photosystem, by increasing non-radiative heat dissipation, could consume the excessive light energy absorbed by PSII. Consequently, the PSII response center is protected from damage by photooxidation and photoinhibition for absorbing excess light energy. In the present study, the level of *qP* declined under NaCl stress, indicating that NaCl stress led to decreased openness of PSII reaction centers. The accumulation of reduced electron acceptors may increase the probability of generating reactive radicals, which may further cause injury to PSII components (Barber and Andersson 1992). The light energy captured from the antenna pigment used for photochemical reactions and the photochemical activity of the PSII reaction center both decreased. Consequently, excess light energy accumulated in the PSII reaction center under NaCl stress. The photosystem was effectively protected by the dissipated excess light energy via the improved level of NPQ. ALA with different concentrations (T1-T4) improved the level of *qP* and decreased the level of NPQ, showing that the salinity stress-induced damage to *C. obtusifolia* had been alleviated by ALA. ALA with concentration of 100 mg·L^-1^ has the opposite effect to NPQ, NPQ improved significantly compared with other concentrations. This result indicated that higher concentration of ALA has significant inhibitory effect to *C. obtusifolia* seedlings.

Increased MDA content is a good reflection of oxidative damage to membrane lipids as well as other such vital molecules as proteins, DNA, and RNA. In the present study, the TBARS levels significantly increased compared with the controls under salinity stress. The peroxidation of membrane lipids may result in enhanced membrane fluidity, which may lead to enhanced electrolyte leakage and support the hypothesis that salinity stress can induce membrane lipid peroxidation. Salinity treatments caused by significantly increased lipid peroxidation during salt stress have been reported. Higher lipid peroxidation has also been reported in salt stress-sensitive rice varieties (Dionisio-Sese and Tobita 1998). Increased lipid peroxidation due to salinity stress results in a significantly increased membrane permeability. The extents of lipid peroxidation and membrane permeability have been used as indices of salt injury and tolerance in Amaranthus (Battacharjee and Mukherjee 1996). Increased membrane permeability has been suggested to reflect the extent of lipid peroxidation caused by ROS (Sairam et al. 1998). In the present study, the TBARS content and membrane permeability increased under NaCl stress. This result indicated that the membrane was damaged by ROS, cell membrane peroxidation occurred, and the normal physiological function of the plasma membrane became disordered. After treatment with exogenous ALA, TBARS content and membrane permeability significantly decreased. Therefore, ALA alleviated the damage caused by NaCl stress.

The accumulations of soluble sugars, soluble protein, and free proline under stress protect plant cells by balancing the osmotic strength of cytosol with that of vacuoles and the external environment (Gadallah 1999). These solutes are cytosolic osmotic substances, and may also interact with cellular macromolecules such as enzymes to stabilize the structure and function of such macromolecules. A direct consequence of a higher osmolyte concentration is the maintenance of comparatively antioxidant enzyme activities (Smirnoff and Cumbes 1989). The results of the present study indicated that the contents of soluble sugar, soluble protein, and free proline treated with ALA in *C. obtusifolia* seedling leaves were significantly higher than those treated with NaCl stress (CK2). A lower osmotic potential within the cell was possibly maintained to help cells absorb water from the external environment, resulting in resistance to the damage caused by NaCl stress.

To endure oxidative damage under conditions of increased oxidative stress such as salinity, plants must possess efficient antioxidant systems. Plants do possess antioxidant systems in the form of enzymes such as SOD, POD, and CAT; they also have an efficient system for decomposing ROS, using the enzyme SOD in chloroplasts (Asada 1999). SOD is located in chloroplasts, mitochondria, the cytoplasm, and peroxisomes. SOD serves as the first line of defense against ROS (Liau et al. 2007). A high SOD activity can efficiently remove O_2_^-^·, which leads to the production of H_2_O_2._ H_2_O_2_ can be scavenged by CAT and GR (glutathione reductase) in the Halliwell–Asada pathway. The accumulation of H_2_O_2_ then begins and exacerbates membrane lipid peroxidation, causing membrane damage. POD could remove SOD disproportionation products and synergizes with SOD, the essential condition of a salt-resistance mechanism. Protective enzymes increase to a high level to remove ROS and keep them at a low level. Consequently, the function and structure of undamaged membranes are maintained. In the current paper, three kinds of antioxidant enzymes significantly increased with different ALA concentrations, although the change trends are slightly different. Apparently, the three enzymes had different strategies for facing stress. Exogenous ALA treatment improved the abilities of the three enzymes for resisting peroxide damage to plant cells.

So far, the reason for ALA-induced increase in antioxidant enzyme activity in plants is still unknown. It may be related to the conversion of ALA into heme, as suggested by a previous study that exogenous ALA processing promotes heme efflux from intact developing cucumber chloroplasts and translates it into hemoglobin (Thoms and Weinstein 1990). ^14^C has also been found to permeate into the porphyrin auxiliary molecules of peroxidase and pigment cells upon treatment with ^14^C-ALA (Van Huyestee 1977). Evidently, heme is an auxiliary component of peroxidase, and ALA treatment promotes the synthesis of heme (Hunter et al. 2005). Consequently, peroxidase activity and anti-oxidative stress are increased.

## Conclusion

The present study revealed that ALA with an appropriate concentration could significantly alleviate NaCl stress-induced damage to *C. obtusifolia* seeds and seedlings. The alleviation is achieved via improved antioxidant enzyme activities, increased Chl content and photosynthetic efficiency, strengthened capacity of scavenging ROS, increased membrane stability, decreased cell osmotic potential, as well as decreased membrane lipid peroxidation.

## Authors’ original submitted files for images

Below are the links to the authors’ original submitted files for images. Authors’ original file for figure 1 Authors’ original file for figure 2 Authors’ original file for figure 3 Authors’ original file for figure 4 Authors’ original file for figure 5 Authors’ original file for figure 6 Authors’ original file for figure 7 Authors’ original file for figure 8 Authors’ original file for figure 9 Authors’ original file for figure 10 Authors’ original file for figure 11 Authors’ original file for figure 12 Authors’ original file for figure 13 Authors’ original file for figure 14 Authors’ original file for figure 15 Authors’ original file for figure 16

## Abbreviations

ALA: 5-Aminolevulinic acid
CAT: Catalase
CHl: Chlorophyll
Fv/Fm: Photochemical efficiency of photosystem II
Fv'/Fm': Photochemical efficiency
GI: Germination index
GR: Germination rate
GV: Germination vigor
NBT: Nitroblue tetrazolium
NPQ: No-photochemical quenching coefficient
POD: Peroxidase
qP: Photochemical quench coefficient
ROS: Reactive oxygen species
SOD: Superoxide dismutase
TBARS: Thiobarbituric acid reactive species
VI: Vigor index
ΦPSII: PSII actual photochemical efficiency.

## References

[CR1] Ahmad S, Wahid A, Rasul E, Wahid A (2005). Comparative morphological and physiological responses of green gram genotypes to salinity applied at different growth stages. Bot Bull Acad Sin.

[CR2] Anu SJ, Rao JM (2001). Oxanthrone esters from the aerial parts of *Cassia kleinii*. Phytochemistry.

[CR3] Asada K (1999). The water-cycle in chloroplasts: scavenging the active oxygens and dissipation of excess photons. Annu Rev Plant Biol.

[CR4] Baker NR, Rosenqvist E (2004). Application of chlorophyll fluorescence can improve crop production strategies: an examination of future possibilities. J Exp Bot.

[CR5] Barber J, Andersson B (1992). Too much of a good thing: light can be bad for photosynthesis. Trends Biochem Sci.

[CR6] Bates LS, Waldran RP, Teare ID (1973). Raipid determination of free proline for water studies. Plant Soil.

[CR7] Battacharjee S, Mukherjee AK (1996). Ethylene evolution and membrane lipid peroxidation as indicators of salt injury in leaf tissues of Amaranthus seedlings. Indian J Exp Biol.

[CR8] Chakrabory N, Tripathy BC (1992). Involvement of singlet oxygen in 5-aminolevulinic acid induced photodynamic damage of cucumber chloroplast. Plant Physiol.

[CR9] Demmig-Adams B, Adams WWIII, Barker DH (1996). Using chlorophyll fluorescence to assess the fraction of absorbed light allocated to thermal dissipation of excess excitation. Physiol Plant.

[CR10] Dionisio-Sese ML, Tobita S (1998). Antioxidant responses of rice seedlings to salinity stress. Plant Sci Limerick.

[CR11] Eiji N, Kensuke K, Mohammad MP (2003). Role of 5-aminolevulinic acid (ALA) on active oxygen-scavenging system in NaCl-treated spinach (*Spinacia oleracea*). J Plant Physiol.

[CR12] Gadallah MAA (1999). Effect of proline and glycine betaine on Vicia faba responses to salt stress. Biol Plant.

[CR13] Genty B, Briantais JM, Baker NR (1989). The relationship between the quantum yield of photosynthetic electron transport and quenching of chlorophyll fluorescence. Biochem Biophys Act.

[CR14] Goel A, Sheoran IS (2003). Lipid peroxidation and peroxide-scavenging enzyme in cotton seeds under natural ageing. Biol Plant.

[CR15] Havaux M, Strasser RJ, Greppin H (1991). Atheoretical and experimental analysis of the *qP* and *qN* coefficients of chlorophyll fluorescence quenching and their relation to photochemical land non photochemical event. Photosynth Res.

[CR16] Heath RL, Packer I (1968). Photoperoxidation in isolated chloroplst I, kinetics and stochiometry of fatty acid peroxidation. Arch Biochem Biophys.

[CR17] Hormann H, Neubauer C, Schreiber U (1994). On the relationship between chlorophyll fluorescence quenching and the quantum yield of electron transport in isolated thylakoids. Photosynth Res.

[CR18] Hotta Y, Tanaka H, Takaoka Y, Takeuchi Y, Konnai M (1997). Promotive effects of 5-aminolevulinic acid on the yield of several crops. Plant Growth Regul.

[CR19] Hotta Y, Watanabe K, Tanaka T, Takeuchi Y, Konnai M (1997). Effects of 5-aminolevulinic acid on growth of plant seedlings. J Pest Sci.

[CR20] Hunter AG, Rivera E, Ferrreira GC (2005). Supraphysiological concentrations of 5-aminolevulinic acid dimerize in solution to produce superoxide radical anions via a protonated dihydropyrazine intermediate. Arch Biochem Biophy.

[CR21] Joshi H, Kapoor VP (2003). *Cassia grandis* Linn. f. seed galactomannan: structural and crystallographical studies. Carbohydrate Res.

[CR22] Kalpana R, Madhava Rao KV (1995). On the aging mechanism in pigeonpea (*Cajanus cajan* L.) seed. Seed Sci. Technol.

[CR23] Korkmaz A (2005). Inclusion of acetyl salicylic acid and methyl jasmonate into the priming solution improves low temperature germination and emergence of sweet pepper seeds. HortSci.

[CR24] Kumar AM, Chaturvedi S, Söll D (1999). Selective inhibition of HEMA gene expression by photooxidation in Arabidopsis thaliana. Phytochemistry.

[CR25] Li Y, Qu JJ, Yang XM, An LZ (2008). A report on ultra-dry storage experiment of *Zygophyllum xanthoxylon* seeds. Bot Stud.

[CR26] Liau YJ, Wen L, Shaw JF, Lin CT (2007). A highly stable cambialisticsuperoxidedismutase from Antrodia camphorata: expression in yeast and enzyme properties. J Biotechnol.

[CR27] Lichtenthaler HK (1987). Chlorophylls and carotenoids: pigments of photosynthetic biomemranes. Methods Enzymol.

[CR28] Lowry OH, Rosebrough NJ, Farr AL, Randall RJ (1951). Protein measurement with the Folin phenol reagent. J Biol Chem.

[CR29] Maxwell K, Johnson GN (2000). Chlorophyll fluorescence-a practical guide. J Exp Bot.

[CR30] McDonald MB (1999). Seed deterioration: Physiology, repair and assessment. Seed Sci Technol.

[CR31] Nishihara E, Kondo K, Parvez MM, Takahashi K, Watanabe K, Tanaka K (2003). Role of 5-aminolevulinic acid (ALA) on active oxygen-scavenging system in NaCl-treated spinach (*Spinacia oleracea*). J Plant Physiol.

[CR32] Noriega GO, Balestrasse KB, Batlle A, Tomaro ML (2004). Heme oxygenase exerts a protective role against oxidative stress in soybean leaves. Biochem Biophys Res Commun.

[CR33] Sairam RK (1994). Effect of moisture stress on physiological activities of two contrasting wheat genotypes. Indian J Exp Biol.

[CR34] Sairam RK, Desmukh PS, Saxena DC (1998). Role of antioxidant systems in wheat genotypes tolerant to water stress. Biol Plant.

[CR35] Sayed OH (2003). Chlorophyll fluorescence as a tool in cereal research. Photosynthetica.

[CR36] Smirnoff N, Cumbes QT (1989). Hydroxyl radicals scavenging activity of compatible isolates. Phytochemisiry.

[CR37] Stobart AK, Ameen-Bukhari J (1984). Regulation of δ-aminolevulinic acid synthesis and protochlorophyllide regeneration in the leaves of dark-grown barley (*Hordeum vulgare*) seedlings. Biochem.

[CR38] Storey R, Wyn-Jones RG (1975). Betaine and choline levels in plnats and their relationship to NaCl stress. Plant Sci Lett.

[CR39] Thoms J, Weinstein JD (1990). Measurement of heme efflux and heme content in isolated developing chloroplasts. Plant Phy.

[CR40] Van Huyestee RB (1977). Porphyrin and peroxidase synthesis in cultured peanut cells. Can J Bot.

[CR41] Von Wettstein D, Gough S, Kananagara C (1995). Chlorophyll biosynthesis. Plant Cell.

[CR42] Wahid A, Parveen M, Gelani S, Basra SMA (2007). Pretreatment of seeds with H_2_O_2_ improves salt tolerance of wheat seedling by alleviation of oxidative damage and expression of stress proteins. J Plant Physiol.

[CR43] Wang LJ, Jiang WB, Zhang Z, Yao QH, Matsui H, Ohara H (2003). 5-Aminolevinilic acid and its potential application in agriculture. Plant Physiol Commun.

[CR44] Xu CC, Zhang JH (1999). Effect of drought on chlorophyll fluorescence and xanthophyll cycle components in winter wheat leaves with different ages. Acta Phytophysiologica Sinica.

[CR45] Xu PL, Guo YK, Bai JG, Shang L, Wang XJ (2008). Effects of long-term chilling on ultrastructure and antioxidant activity in leaves of two cucumber cultivars under low light. Physiol Plant.

[CR46] Yemm EW, Willis AJ (1954). The estimation of carbohydrates in plant extracts by anthrone. Biochem J.

[CR47] Zhang CP, He P, Du DD, Wei PX, Yu ZL, Xie YZ, Liu HY (2011). Effect of exogenous Nitric Oxide donor SNP on seed germination and antioxidase activities of *Perilla frutescens* seedlings under NaCl stress. JChinese Med Materials.

[CR48] Zhang CP, He P, Du DD, Yu ZL, Hu SJ (2010). Effect of zinc sulphate and PEG priming on ageing seed germ ination and antioxidase activities of *Perilla frutescens* seedlings. China J Chinese Materia Medica.

[CR49] Zhang SR (1999). A discussion on chlorophyll fluorescence kinetics parameters and their significance. Chinese Bulletin of Botany.

[CR50] Zhang ZJ, Li HZ, Zhou WJ, Takeuchi Y, Yoneyama K (2006). Effect of 5-aminolevulinic acid on development and salt tolerance of potato (Solanumtuberosum L.) microtubers in vitro. Plant Growth Regul.

