# Supplementary figures and images for: Role of 5-aminolevulinic acid in the salinity stress response of the seeds and seedlings of the medicinal plant Cassia obtusifolia L
Source: Bot Stud. 2013 Aug 23;54:18. doi: 10.1186/1999-3110-54-18 (PMC5430315; doi:10.1186/1999-3110-54-18)

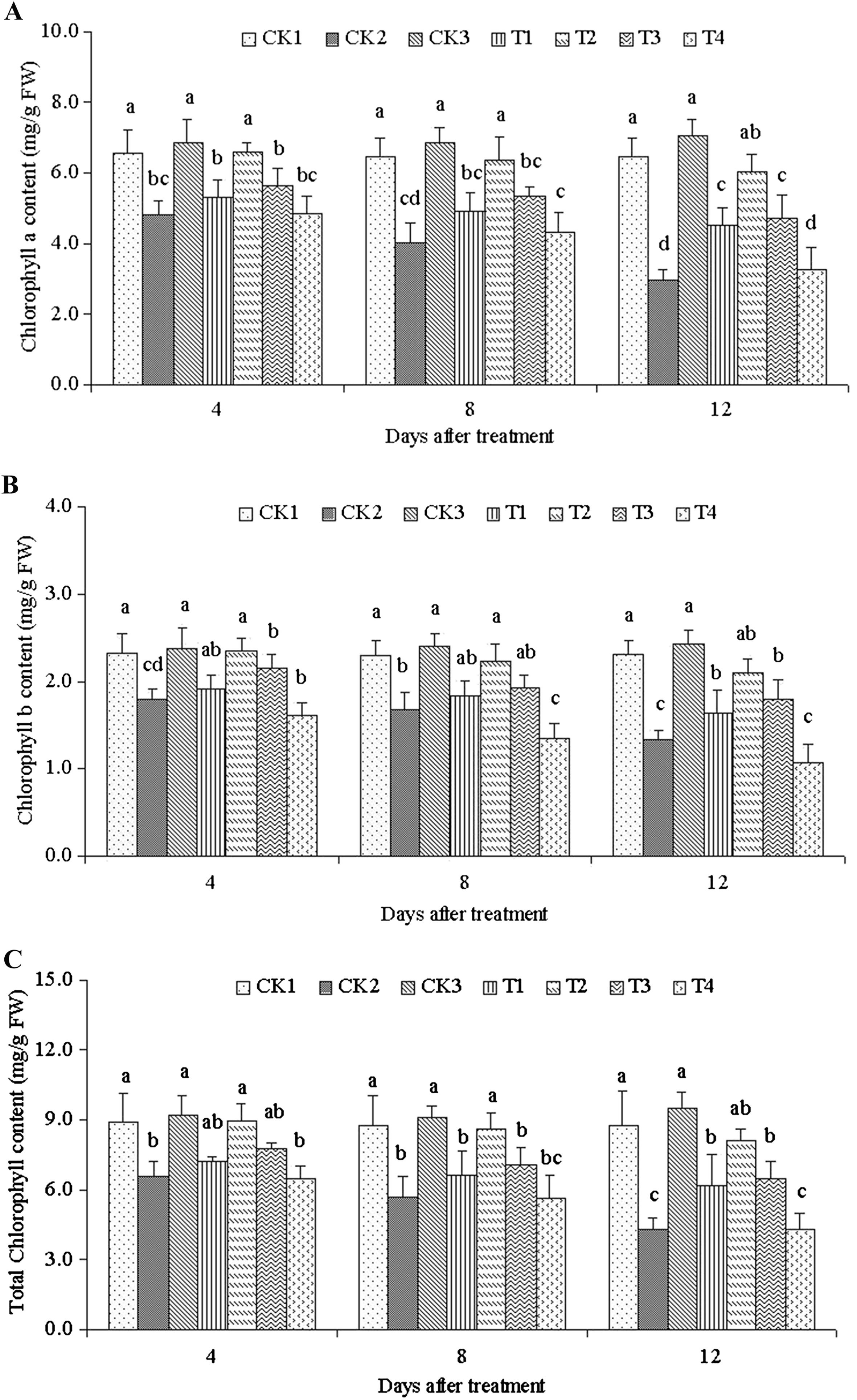

Supplement: Supplementary file 1 — Authors’ original file for figure 1 [file 40529_2011_24_MOESM1_ESM.tiff]

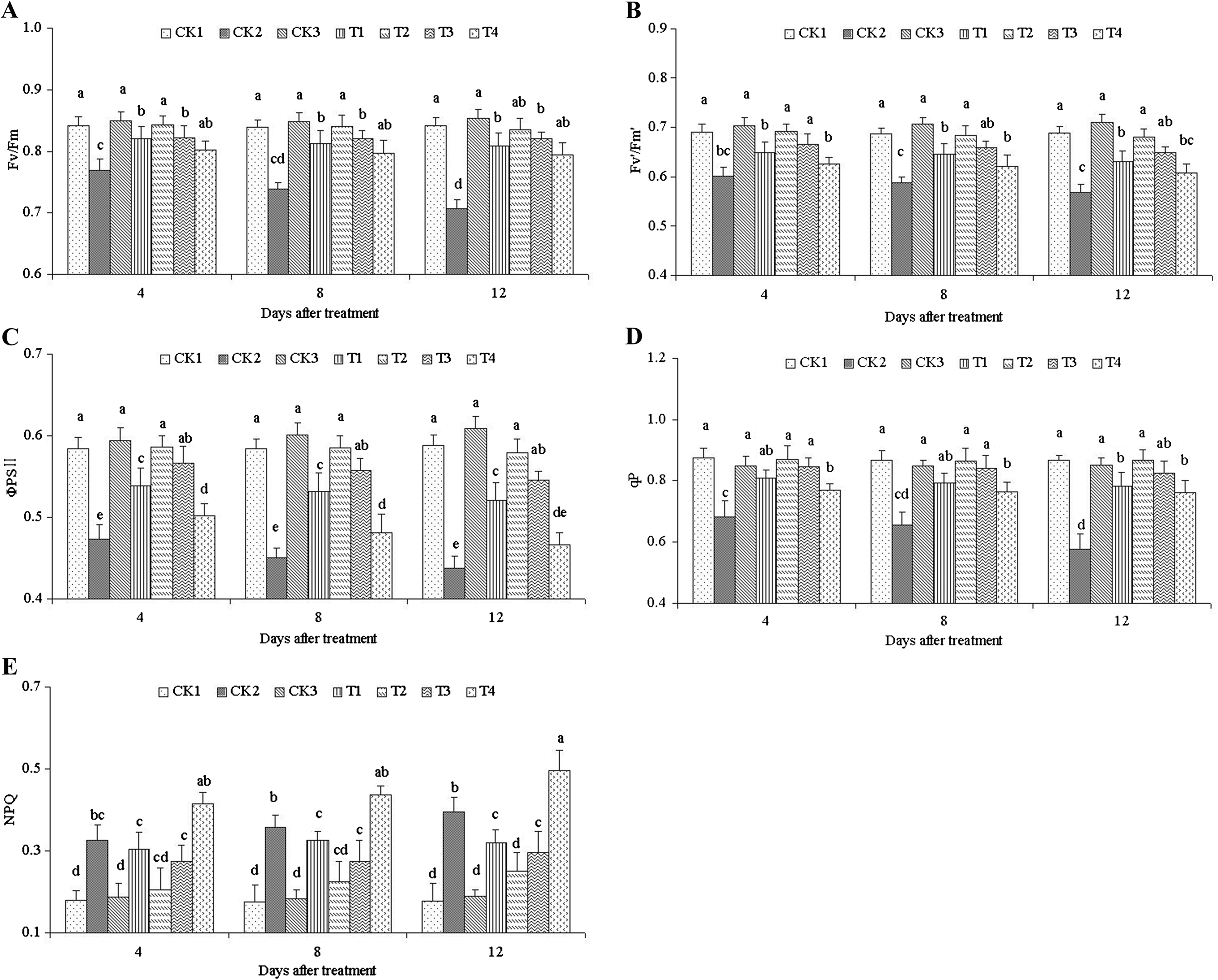

Supplement: Supplementary file 2 — Authors’ original file for figure 2 [file 40529_2011_24_MOESM2_ESM.tiff]

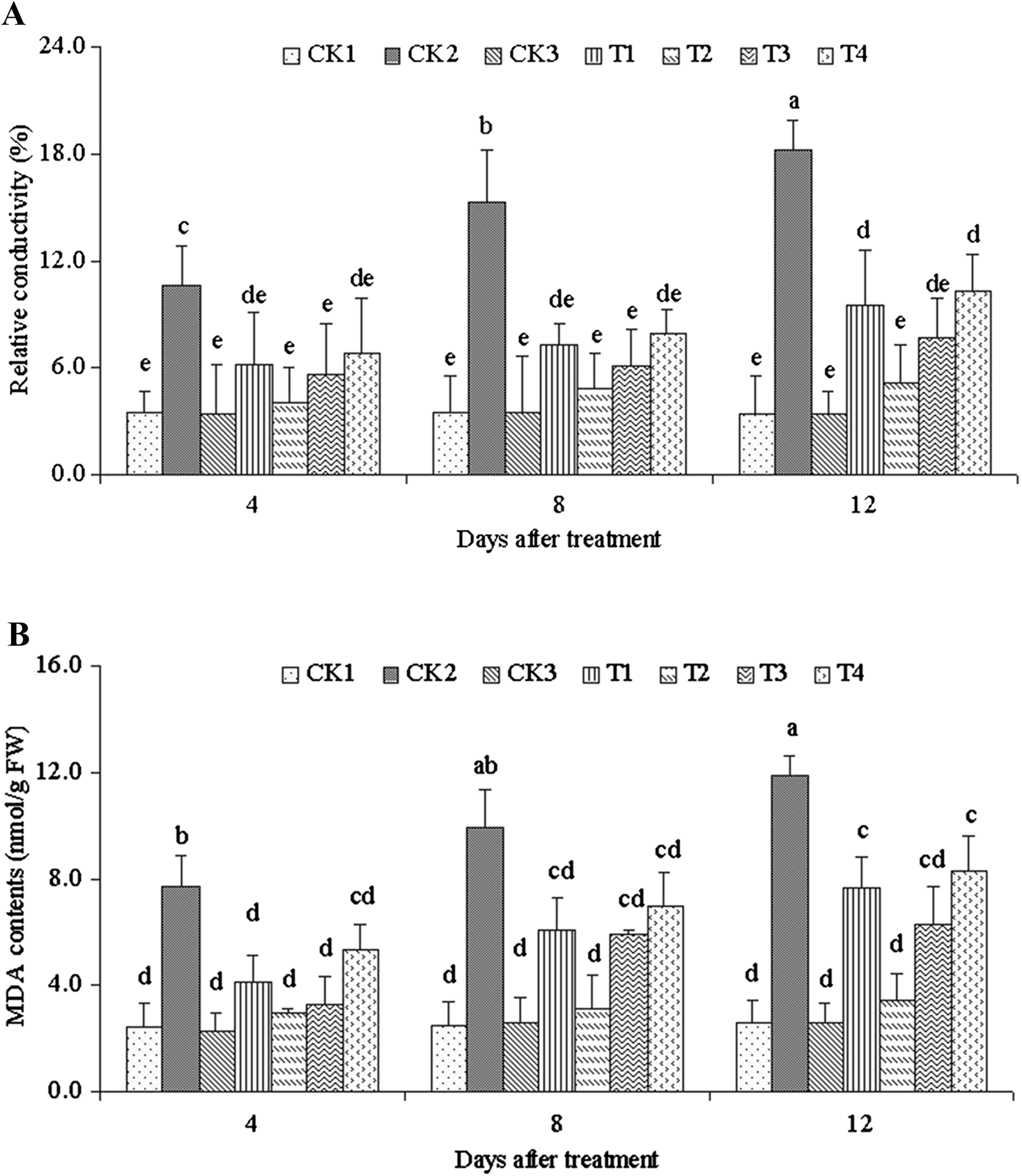

Supplement: Supplementary file 3 — Authors’ original file for figure 3 [file 40529_2011_24_MOESM3_ESM.tiff]

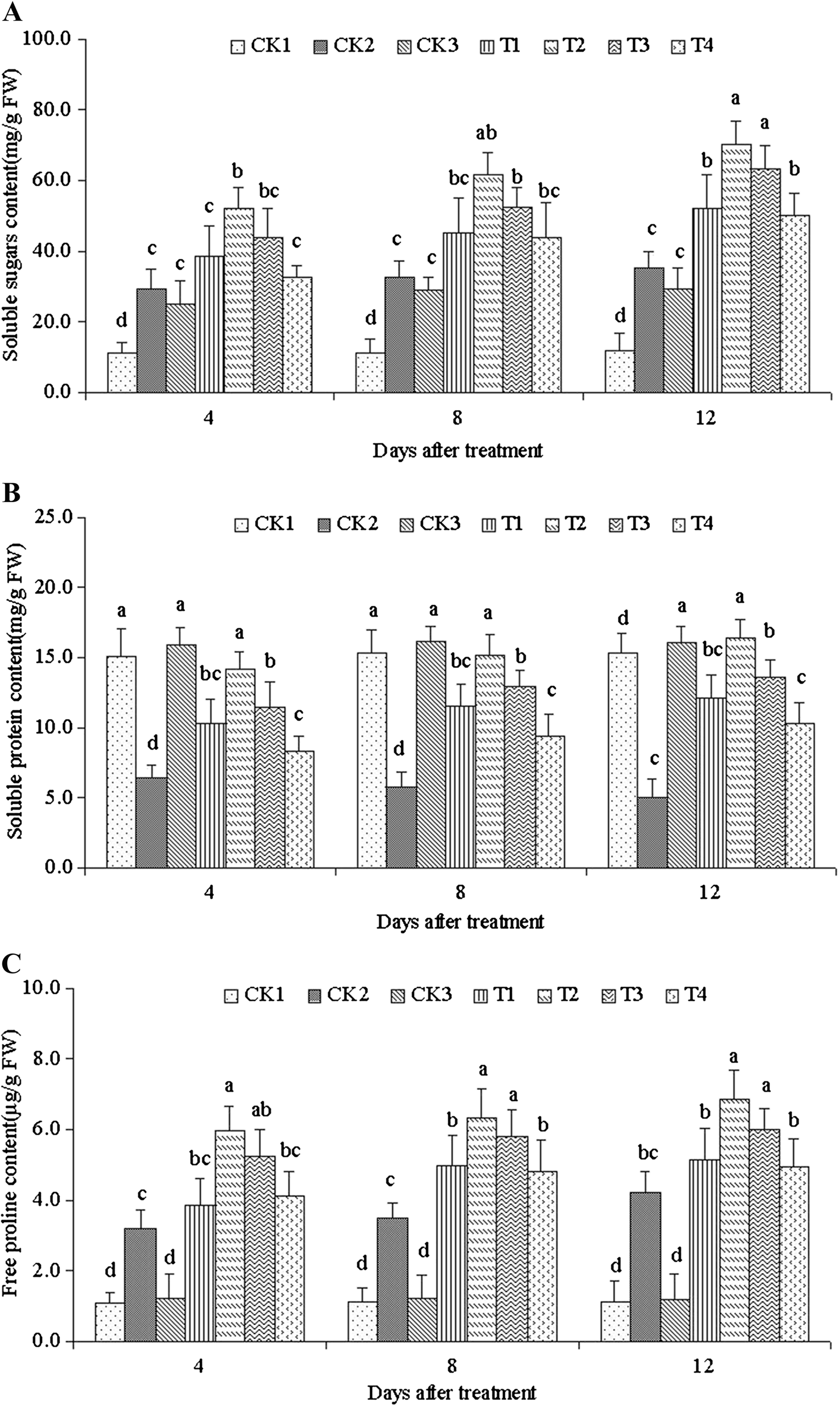

Supplement: Supplementary file 4 — Authors’ original file for figure 4 [file 40529_2011_24_MOESM4_ESM.tiff]

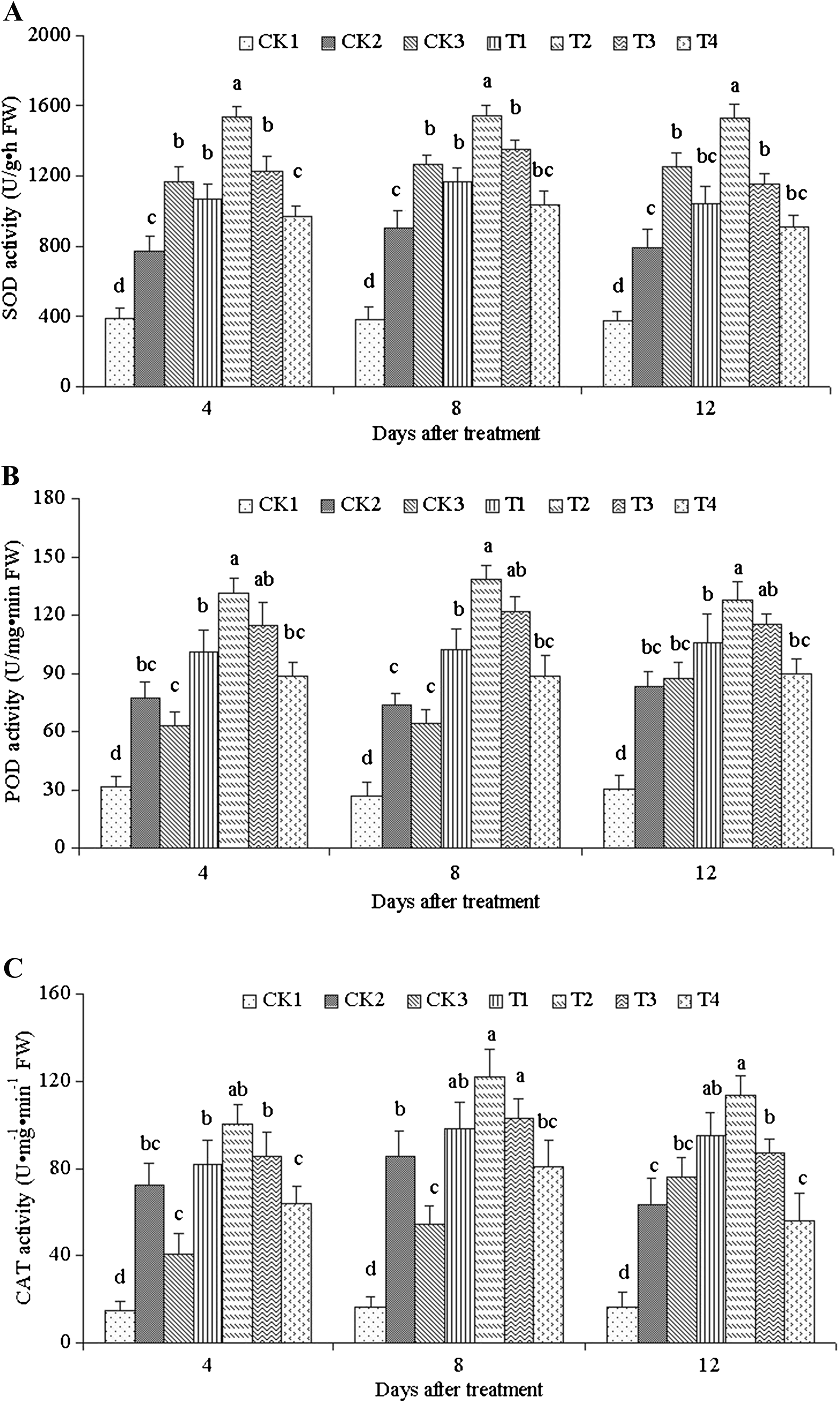

Supplement: Supplementary file 5 — Authors’ original file for figure 5 [file 40529_2011_24_MOESM5_ESM.tiff]

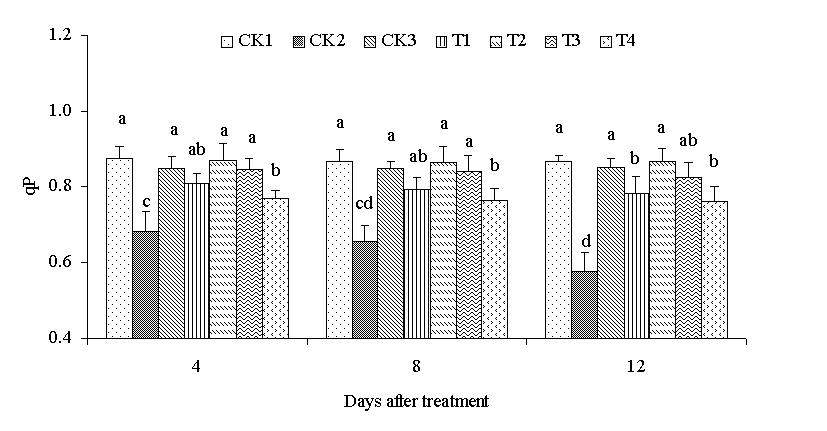

Supplement: Supplementary file 7 — Authors’ original file for figure 7 [file 40529_2011_24_MOESM7_ESM.jpeg]

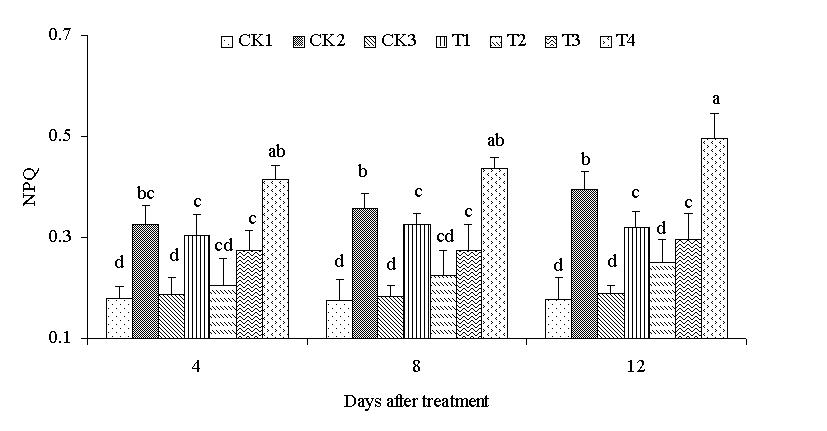

Supplement: Supplementary file 8 — Authors’ original file for figure 8 [file 40529_2011_24_MOESM8_ESM.jpeg]

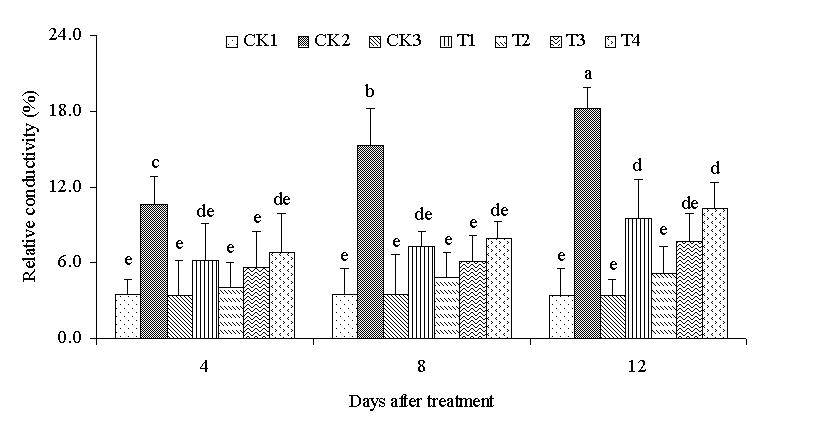

Supplement: Supplementary file 9 — Authors’ original file for figure 9 [file 40529_2011_24_MOESM9_ESM.jpeg]

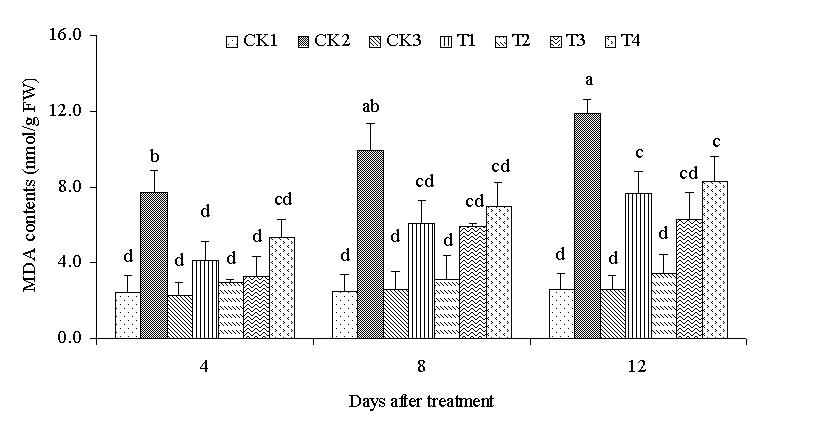

Supplement: Supplementary file 10 — Authors’ original file for figure 10 [file 40529_2011_24_MOESM10_ESM.jpeg]

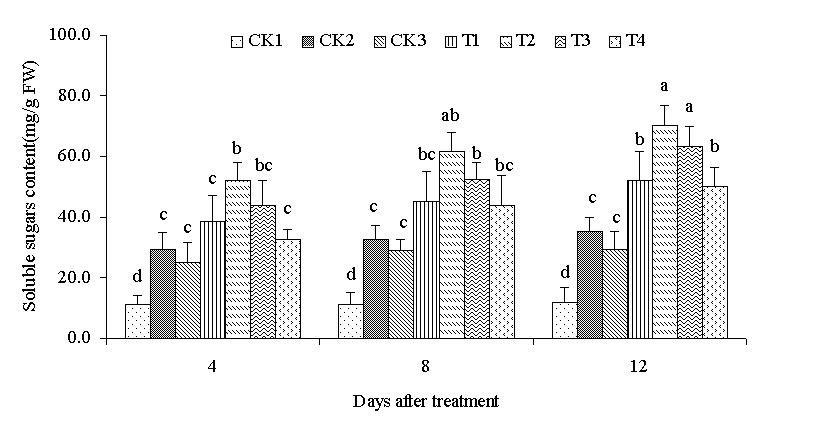

Supplement: Supplementary file 11 — Authors’ original file for figure 11 [file 40529_2011_24_MOESM11_ESM.jpeg]

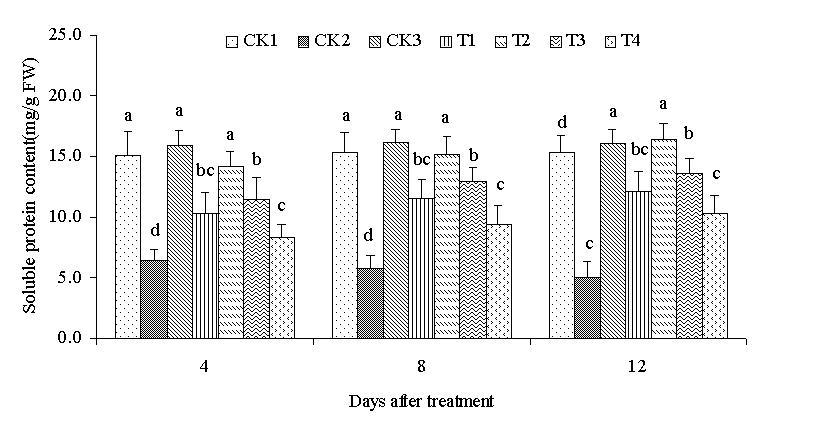

Supplement: Supplementary file 12 — Authors’ original file for figure 12 [file 40529_2011_24_MOESM12_ESM.jpeg]

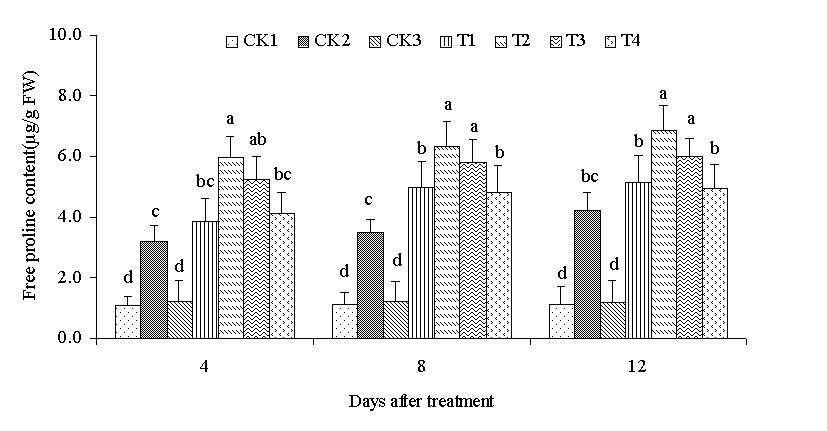

Supplement: Supplementary file 13 — Authors’ original file for figure 13 [file 40529_2011_24_MOESM13_ESM.jpeg]

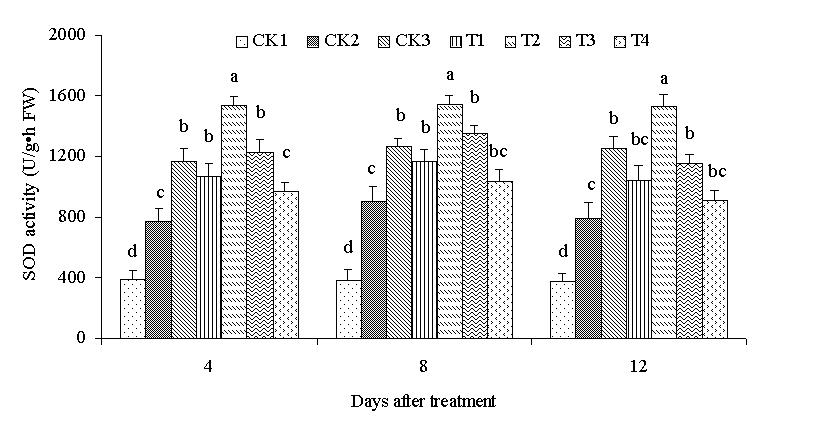

Supplement: Supplementary file 14 — Authors’ original file for figure 14 [file 40529_2011_24_MOESM14_ESM.jpeg]

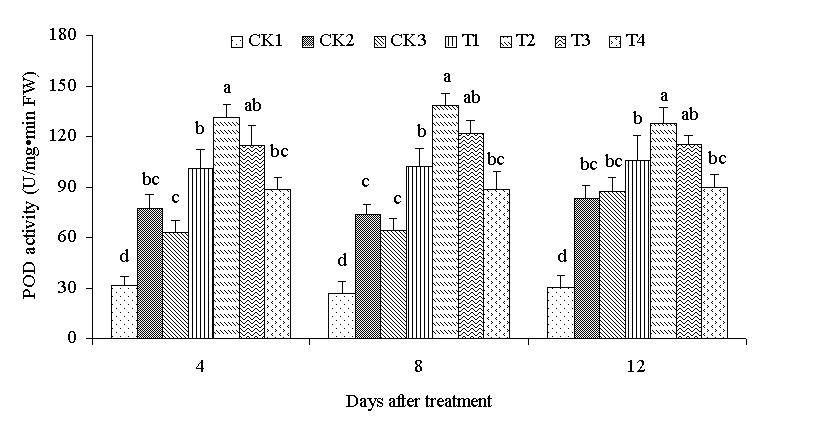

Supplement: Supplementary file 15 — Authors’ original file for figure 15 [file 40529_2011_24_MOESM15_ESM.jpeg]

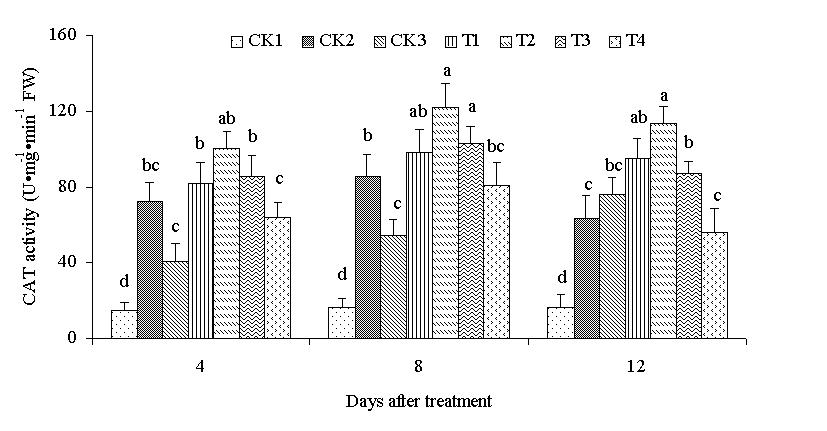

Supplement: Supplementary file 16 — Authors’ original file for figure 16 [file 40529_2011_24_MOESM16_ESM.jpeg]
